# Reflective practice across speech and language therapy and education: a protocol for an integrative review

**DOI:** 10.12688/hrbopenres.13234.2

**Published:** 2022-03-24

**Authors:** Jessica McCluskey, Aoife L. Gallagher, Carol-Anne Murphy

**Affiliations:** 1 School of Allied Health, University of Limerick, Castletroy, Limerick, V94 T9PX, Ireland; 2 Health Implementation Science and Technology Cluster, Health Research Institute, University of Limerick, Limerick, Ireland

**Keywords:** Reflection, reflective practice, Speech and Language Therapist, speech language and communication needs, teacher, education, collaboration, inclusion

## Abstract

Effective co-practice is considered a linchpin of inclusive education. Speech and language therapists (SLT), in collaboration with teachers, are amongst the professionals who have a role in ensuring inclusion for students. The challenges of collaboration are well documented, with communication considered a potential antidote. Proposals for how collaborative communication can take place often align with models of reflection. Uncertainty around a shared language for reflection within and across the professions of teaching and SLT may pose a barrier to it occurring. Reflection has long been documented as a strategy used by effective clinicians to improve practice. Hence, teachers and SLTs reflecting together could be considered ‘a port of entry’ for effective collaborative practice. This study aims to synthesise literature and knowledge on the phenomenon of reflective practice across the professions to facilitate collaboration for inclusive education.

The method of qualitative evidence synthesis will be an integrative review. A systematic search will be conducted to extract empirical studies, reviews and theoretical papers on the topic of reflection across both professions.  An adapted version of the PRISMA reporting guidelines will be used in the development, design and reporting of this review. Four databases will be searched: CINAHL, SCOPUS, Education Source and ERIC. A web-based search will also be conducted to retrieve relevant policy documents. Included literature will be appraised using the M-MAT and an adapted checklist from the Joanna Briggs Institute. Deductive content analysis will endeavour to determine if a shared language exists about reflection, across the professions of teaching and speech and language therapy.

Establishing a shared language represents a first step towards the development of a framework for collaborative reflection between teachers and SLTs. This is turn serves to inform future research, policy and practice regarding how speech and language therapist can work collaboratively with teachers in schools.

## Introduction

The need to work towards inclusive education for all was recognized internationally with the publication of the Salamanca Statement ( UNESCO, 1994). Effective co-practice between a variety of professionals is proposed as a means by which inclusive education can be achieved and is a term used generally in situations where staff from different professions work together ( McKean *et al*., 2017). In professional policy ( ASHA, 2001; RCSLT, 2018; SPA, 2011) and the literature ( Law *et al.*, 1999; McCartney, 1999; McCartney, 2000; McCartney, 2002) speech and language therapists (SLT) are recognised as some of the professionals who play a crucial role in the implementation of practical and strategic changes, to ensure inclusion is the norm for students with speech, language and communication needs (SLCN) in schools ( Ebbels *et al*., 2019; McKean *et al*., 2017). The response to this in some countries has been the provision of SLT services as part of the education system; however, the specifics of facilitating effective collaboration between SLT’s and teachers working together in practice remains limited ( Gallagher *et al*., 2019). What is clear, is that co-practice is challenging, and it is hard to work together, particularly in inter-professional contexts.

Literature from the 1990s onwards outlines what makes co-practice so difficult. Developing reciprocal relationships ( Field, 2003), establishing shared language ( Law *et al*., 2001) and managing power dynamics are just some of the factors that can influence the effectiveness of co-practice between individuals. Another challenge, unique to the therapeutic and pedagogical co-practice context, is the many forms that co-practice in a school can take. Suleman *et al.* (2014) has proposed frameworks that detail the ways in which teachers and SLT can work together, classifying a transdisciplinary model as the most integrative. Transdisciplinary co-practice is distinctive from others due to the professional overlap ( Hall & Weaver, 2001) in addition to the sharing of roles and responsibilities. Striving to work together effectively in this way requires the recognition of a potential threat to professional identity ( Binyamin, 2017).

Communication is proposed as a potential solution to the challenges experienced. In the literature, this communication is characterised by an ongoing dialectal process ( Bleakley & Bligh, 2008) that facilitates careful consideration of self, others, experiences and changes ( Mackey, 2007). Aligned with Mackey’s proposal of ‘careful consideration’ are definitions of reflection and models of reflective practice. Boud *et al*. (1985) stated that reflection is ‘a conscious activity’ that can be engaged with to explore experience and understand better. “Reflective practice is the act of reflection, captured in some way (discussion, writing), on a systematic basis” ( CIPD, 2020:3). It is a requirement for the development of new skills as well as developing the responsive capacity of a person to create productive relationships, manage emotions, make decisions and cope with stress ( CIPD, 2020). It is therefore considered particularly useful in dealing with difficult or challenging situations ( Moon, 1999). Models and frameworks exist that facilitate reflective practice in professional contexts. For example, Dr Stephen Brookfield’s Lens Theory (1995) is frequently cited in relation to teaching as a means of supporting the professional reflection of teachers, while the Reflective Theory Model of Argyris & Schӧn (1978) has been quite influential in the field of SLT ( Caty *et al*., 2015). Reflective practice is regarded as essential to the professional role ( Finlay, 2008). Despite its centrality to both professions there has been remarkably little research into the reality of how it is used by practitioners ( Bray, 2020). Publications in the fields of nursing, teacher education, social care as well as across other medical professions ( Boud, 2010) most often refer to the use of reflective practice with higher education students or as part of CPD.

As outlined, integrative co-practice between SLTs and teachers in schools continues to be novel and can pose a threat to professional identity. Finlay stated generally that engaging in reflective practice often forms the “bedrock of professional identity” (2008:3). Thus, the use of reflection by teachers and SLTs may be valuable in the attempt to forge new identities within a novel working context.

A potential difficulty is that across different disciplines and intellectual traditions, what is understood by ‘reflective practice’ varies considerably ( Fook *et al*., 2006). In order to facilitate collaboration of any kind, there is a need for a shared language ( Law *et al*., 2001). Therefore, a shared or common language for reflection must be identified if successful co-reflection between the two professions is to be considered. Reflecting together is not only required to ensure effective co-practice but also to facilitate the transition from an individual profession working comfortably in their own field to an integrative team member working clinically in the educational context. However **, “**the gap between the ‘high ground’ of theory and the ‘swampy lowlands’ of practice” ( SkillsYouNeed, 2020) remains relatively unexplored. An initial overview of literature, policy and practice would indicate that thus far methods for reflecting together, outside of the IPE context, are at best underutilised if not completely underdeveloped. There therefore exists a need for exploration, with the literature as the first point of reference. By interrogating the literature in this research study, we hope to determine if a shared understanding of reflective practice exists between health and education. 

The question guiding this research is: How is reflection understood across the professions of SLT and teaching?

The principle aim of this study is to synthesise literature and knowledge on the phenomenon of reflective practice across the professions of speech and language therapy and teaching. This will be achieved by addressing four objectives:

(i) What is common or shared about the understanding of reflective practice across both professions?
(ii) What is different about the understanding of reflective practice across both professions?
(iii) What implication do these commonalities and differences have for collaborative practice between SLTs and teachers?
(iv) What remains unknown or needs to be investigated for collaborative reflection between the professions?

Addressing these objectives will provide insight into the value placed on reflection by both professions as well as determining the language and terms used to describe reflection and reflective practices across disciplinary contexts. New knowledge from this research can be used to inform the development of a framework to guide collaborative reflective practice between teachers and SLTs. This knowledge will be relevant to those in practice, research, management and policy development, working towards truly collaborative interdisciplinary relationships as part of an inclusive education system.

## Protocol

This protocol is reported in line with the Preferred Reporting Items for Systematic review and Metal-Analysis Protocols (PRISMA-P) checklist ( McCluskey *et al*., 2021). An integrative review ( Kirkevold, 1997) has been chosen as the method for this study. This is a method that allows the integration of different types of knowledge about practice-based questions. It involves identifying, analysing and synthesising a diverse range of literature sources; empirical, theoretical and policy. It is considered an effective method to guide evidence informed practice ( Crawford & Rondinelli, 2013). In this study we propose to use Souza *et al.*’s (2010), framework for conducting integrative reviews. This framework incorporates six phases: preparing the guiding question, searching and sampling the literature, data collection, critical analysis of the studies included, discussion of results and finally the presentation of the integrative review. Suri (2019) outlined ethical considerations when conducting a systematic review, which will also be contemplated across all six phases of this integrative study.

### Preparing the guiding question

The research question and objectives above have been created based on practice knowledge and exploration of the literature. The question is specific to speech and language therapy and education in order to capture the literature pertaining to reflective practice in these fields only. Identification of additional research questions and objectives may be an iterative process, informed by continued immersion in the literature while conducting this review.

### Searching and sampling

***Sampling strategies.*** The first search strategy will be a systematic search of published, peer-reviewed literature. A comprehensive, systematic search will be conducted to extract empirical studies, reviews and theoretical papers from electronic databases. The choice of databases was informed by preliminary searches and will be confined to CINAHL, SCOPUS, Education Source and ERIC. In order to ensure key literature is not missed, noteworthy references from papers retrieved will also be traced using a snowballing ( Webster & Watson, 2002) technique. Literature will also be obtained by hand searching reference lists of included articles. The second search strategy will be a manual web-based search. This search will include the websites of professional organisations and publications from government-based reports and guidelines. This will ensure a comprehensive sample of current policy documents is obtained. The web-based search will retrieve a more purposive sample of online literature and will follow systematic procedures as outlined in Stansfield *et al.* (2016). Seminal literature and policy known to the researchers will also be included if not retrieved through the two search strategies.

***Search criteria.*** To support the development of search terms and the criteria to screen studies the SPIDER ( Hewitt-Taylor, 2017) (Sample, Phenomenon of Interest, Design, Evaluation, and Research type) framework was used due to its specificity ( Methley *et al*., 2014). Search terms were generated based on keywords determined by team members, and further developed by an overview of literature on the topics of reflective practice, collaboration between SLT and teachers as well as from educational policy in Ireland. Alongside the specific database headings, Medical Subject Headings (MeSH) and a thesaurus were also used. The table below also outlines the inclusion and exclusion criteria using the SPIDER headings. Depending on papers retrieved, criteria for inclusion and exclusion may need to be adjusted ( Levac *et al*., 2010).

### Selection process

A sample initial search string is shown in Table 2 to guide the electronic database search. This string will continue to be developed iteratively as the search progresses ( Levac *et al*., 2010). Once the electronic database, web-based search strategies have been completed snowballing techniques will be applied. To support the clear reporting of the search strategy procedure, an adapted version of the PRISMA statement (Preferred Reporting Items for Systematic Reviews and Meta-Analysis Protocols) ( Moher *et al*., 2009) will be used to represent the integrative review process.

The final citations and literature will be uploaded to Endnote software and the duplicate citations removed. Database literature will be screened first by two reviewers (JM; first author & BF; therapists from the practice context) for title and abstract, and subsequently, by full-text review ( Cooke *et al*., 2012) in line with the study selection criteria detailed in Table 1. Some of this literature will be double screened (20%), both by title and abstract as well as full text, to ensure the transparency of the study selection process. If opinions differ regarding the inclusion of any screened literature a discussion will take place to establish consensus. A third reviewer (AG; second author) will also be available to help facilitate a final decision if an agreement cannot be reached. All steps and decisions related to this process will be documented.

**Table 1.** Search terms.

	Provisional Search Terms	Inclusion Criteria	Exclusion Criteria
Sample	speech therapy, speech and language therapy, speech pathology, speech and language pathology, speech and language pathologist, speech and language therapist, speech therapist teacher, primary school teacher, post-primary school teacher, secondary school teacher, kindergarten teacher, middle school teacher, high school teacher, teaching, educator, education	Speech and language therapists, student therapists and fully qualified Educators; primary and/or post primary teachers preservice or fully qualified.	Other allied health professionals Early years providers, lecturers, school support staff, speech and language therapy assistants who have engaged in reflection or reflective practice.
Phenomenon of Interest	Reflection, mental process, reflective practice, personal reflection, self-review, self-awareness, self- critique, self-appraisal, self-analysis, intra-personal awareness, personal cognizance reflective dialogue, critical evaluation	Empirical studies must involve teachers and/or Speech and Language therapists engaging in reflective practice either independently, within their discipline or in a transdisciplinary way. Theoretical literature and policy must pertain to reflection or reflective practice wholly or in part.	Reflection of other disciplines or professions Reflection of clients or those being educated Reflections of parent/guardians of those engaged in SLT or education.
Design	n/a	Empirical studies; qualitative, quantitative and mixed methods Systematic reviews; secondary research Theoretical papers	
Evaluation	n/a	Theory on reflection in the fields of SLT or education, experience and/or feelings of sample population when engaged in reflection, impact or outcomes of engaging in reflection, guidance on reflection from professional websites, policy and resources.	Reflection on the history of the profession.
Research type	n/a	Peer reviewed journals Full text In English Published: no date limit -2021 Policy from professional bodies of teachers and speech therapists.	Thesis Dissertations Opinion pieces Protocols Editorials Policies on reflection or reflective practice not related to teaching or speech and language therapy

**Table 2.** Search string.

Search number	Search strategy
S1	speech therapy OR speech and language therapy OR speech pathology OR speech and language pathology OR speech and language pathologist OR speech and language therapist OR speech therapist OR Teacher OR primary school teacher OR post-primary school teacher OR secondary school teacher OR kindergarten teacher OR middle school teacher OR high school teacher OR teaching, educator OR education
S2	Reflect* OR reflection OR mental processes OR reflective practice OR personal reflection OR self-review OR self-awareness OR self-critique OR self-appraisal OR self-analysis OR intra-personal awareness OR personal cogni#ance OR reflective dialogue OR critical evaluation
S3	S1 + S2

### Quality appraisal and data extraction

The final papers will be read in-depth several times by the first author (JM) to facilitate initial data extraction. The date of the study, author(s) as well as the sample population or context will be extracted from all included literature. Additional data extracted will vary depending on the literature type; empirical, review study, theoretical etc. For example, data extracted from theoretical literature will focus on the theories or models of reflection referenced, constructs and pathways of these theories and disciplinary origins. Data extracted from empirical literature may include information about the study design and a description of how reflection was be used. To support this process, data extraction frameworks for each literature type will be developed based on those proposed by the Joanna Briggs Institute and trialled on 5–6 papers and pieces of policy before applying broadly to all literature included in this study.

Literature included in the final synthesis will also be appraised for quality. The appraisal tool selected will be determined by the literature, likely calling for the use of different tools. The mixed method appraisal tool (M-MAT) ( Hong *et al*., 2018) will be used to assess the methodological quality of empirical research. This is a validated tool that allows for the appraisal of qualitative, quantitative and mixed-method studies ( Gallagher *et al*., 2019). The quality of theoretical literature will be ascertained/appraised using a tool based on the ‘Joanna Briggs Institute (JBI) Checklist for ‘text and opinion’ ( Johanna Briggs Institute – Critical Appraisal Tools).

All literature will be critically appraised independently by two reviewers. Appraisal will give consideration to how the interests of different stakeholders are represented in the literature. Reviewers will also recognise the importance of critically reflecting on the contextual positioning of the authors of primary research being analysed. Appraisal decisions will be recorded and the reason for exclusion reported. Where there is a lack of agreement, the involvement of a third reviewer will be called upon. Studies which do not adequately meet the appraisal criteria will be discarded.

### Analysis

Content analysis, using the Nvivo computer software, will be used to analyse the extracted data in an attempt to describe and quantify the phenomenon ( Downe-Wamboldt, 1992; Krippendorff, 1980; Sandelowski, 1995) of reflection across the speech and language therapy and educational contexts. To facilitate iterative comparison and interpretation, data extracted will be reduced in accordance with a reflection framework. While theories about the phenomenon of reflection, and frameworks for its application, exist in both the educational and speech therapy contexts, little or fragmented knowledge exists about reflection occurring between these professionals when working together. In the selection of a framework, the author considered the context in which reflection between teachers and SLTs is most likely to occur. Hence, the lenses of reflection proposed by Stephen Brookfield often used in educational contexts, will guide analysis.

Within his Lens theory, Brookfield (1995) promotes reflecting on practice (work/life experiences) through other lenses, not only from our own perspective but from multiple perspectives, including reflecting on theory. Brookfield also calls attention to the importance of emotions in the process of reflection, which is a limitation identified in the work of earlier theorists. However, Lens Theory is not without its own limitations. It does not take into consideration the context of the practitioner and it lacks the explicit link of reflection for future. Brookfield also appears to see all teaching and learning taking place in the classroom and does not consider the more informal settings for teaching and learning. As collaboration between the professions of SLT and teachers takes place primarily in the educational setting, the concepts or lenses of Brookfield’s framework will be used to create a strict categorisation matrix for deductive analysis. This framework will be trialed on a number of papers initially, before applying to the final collection of literature, which will then be reviewed for content and coded for reference to these lenses in the description of or engagement with reflection. There is the potential that new concepts could be derived from the data due to the novel nature of considering co-reflection between teachers and SLT. The data that do not fit with Brookfield’s lenses will also be considered, with a view to creating new concepts using an inductive analysis process.

It is anticipated that the results from this analysis will be represented in a visual way. This visual representation will depict what is shared and what is different about how SLTs and teachers reflect. It is predicted that this visual will either build upon the framework of reflection posed by Stephen Brookfield or will propose a new framework for collaborative reflection within the educational context. The development of a new or adapted framework will also allow for navigation of some of the identified limitations to Lens Theory.

Addressing rigour will be an essential aspect when conducting this integrative review. To ensure this research can be considered reliable, detailed descriptions of the literature search, quality appraisal and analysing process will be given when reporting results along with the use of appendices and tables to demonstrate links between the data and results ( Polit & Beck, 2004). The researcher will also be aware of issues related to combining empirical and theoretical reports. During analysis, consideration will be given to how the primary researcher’s contextual positioning (an SLT working in education) may influence the understanding derived from evidence in the literature. Any unavoidable bias will be acknowledged in the limitations of the final study.

## Discussion

From reviewing the literature to date, it appears that this will be the first integrative review seeking to synthesise what is shared and what is different in terms of how speech and language therapists and educators engage with reflection in their practice. The findings of this review will be relevant for researchers, practitioners and policymakers working towards truly collaborative relationships in education. This review will also contribute to the growing body of literature regarding SLT and teachers working in collaboration with those who have SLCN ( Ebbels *et al*., 2019; Gascoigne, 2006; McCartney, 2002; McKean *et al*., 2017; Starling *et al*., 2012). It also seeks to contribute to the literature surrounding best practice for inclusive education by developing a framework for collaborative reflection. Adding to the research knowledge in this area will simultaneously reduce research waste by synthesising and conceptualising available evidence that can be used to describe the phenomenon of reflection amongst and between these professionals.

### Presentation of findings

In order to disseminate the findings from this research, a review paper will be written for publication in a peer reviewed journal such as *Journal of Language, Speech and Hearing Services in Schools (LSHSS)* or the *Journal of Interdisciplinary Studies in Education (JISE)*. Opportunities to disseminate at conferences will also be sought providing an opportunity to share knowledge and findings on this topic with a range of professionals, outside of speech and language therapy. Conferences may also provide the opportunity to network with teachers and practitioners to establish the value other professions place on collaborative reflection as a way to enhance practice for inclusive education.

### Study status

The research question, aims and objectives are now soundly developed, along with the research design. Search terms have been developed as illustrated in this protocol and will continue to be iteratively developed throughout the literature search phase. Inclusion and exclusion criteria have been determined for literature retrieved. Similar to the development of search terms, inclusion and exclusion criteria will be informed by the search process. Methods for appraising literature have been noted, as well as methods for extraction, analysis and data synthesis. The database and web-based search phases of this research are almost complete. Screening and appraisal of literature will shortly commence followed by data extraction.

## Data availability

No data are associated with this article.

### Reporting guidelines

Figshare: PRISMA-P Checklist_McCluskey *et al.* 2021. https://www.doi.org/10.6084/m9.figshare.14170169.v1 ( McCluskey *et al*., 2021).

Data are available under the terms of the Creative Commons Attribution 4.0 International license (CC-BY 4.0).
