# Supplementary material for: Reflective practice across speech and language therapy and education: a protocol for an integrative review
Source: HRB Open Res. 2022 Mar 24;4:29. Originally published 2021 Mar 22. [Version 2] doi: 10.12688/hrbopenres.13234.2 (PMC8591512; doi:10.12688/hrbopenres.13234.2)
Supplement: Peer review history

# Peer review: Reviewer response for version 2

**DOI:** 10.21956/hrbopenres.14770.r31720

**Published:** 2022-04-07

**Authors:** Paula Zwozdiak-Myers

**Affiliations:** 1 School of Education, Brunel University London, London, UK

More than happy to amend my previous recommendation to Approved status based on the authors response to feedback suggestions within their revision. Nothing more I would like to add at this stage.

Is the study design appropriate for the research question?

Yes

Is the rationale for, and objectives of, the study clearly described?

Yes

Are sufficient details of the methods provided to allow replication by others?

Yes

Are the datasets clearly presented in a useable and accessible format?

Not applicable

Reviewer Expertise:

Reflective practice for professional development in teacher education; Social justice, equity and inclusion; Ethics in educational research

I confirm that I have read this submission and believe that I have an appropriate level of expertise to confirm that it is of an acceptable scientific standard.

# Peer review: Reviewer response for version 1

**DOI:** 10.21956/hrbopenres.14396.r30647

**Published:** 2021-11-11

**Authors:** Rena Lyons

**Affiliations:** 1 Discipline of Speech and Language Therapy, National University of Ireland Galway, Galway, Ireland

This study protocol is well thought through and written. It is an exciting piece of research and scientifically sound. It will contribute to the knowledge base and hopefully help to enhance collaborative practice. 

 There are a few minor issues that could be addressed to strengthen the protocol:

1. In the abstract the authors mention that 'the method of qualitative evidence synthesis will be an integrative review'. This is a bit confusing. Qualitative evidence synthesis is a method in and of itself and in the Protocol itself it is clear that an integrative review will be conducted. Further clarification is required.
2. In the Introduction, the authors outline some barriers to co-practice in paragraph two - I wonder is it worth also mentioning other barriers beyond relationships, shared language and power dynamics such as culture differences and more 'meso' level barriers. Even though this protocol will be focusing more on the individual level, it is worth acknowledging the wider context too.
3. In paragraph 3 of the Literature Review, the authors argue that communication is a potential solution - do they mean reflective practice is a potential solution? Or could the authors elaborate on what communication means in this context? 
4. There a few small typos that could be addressed e.g., therapy missing on page 3 (in the second last paragraph) and on page 4 should be Meta-Analysis.
5. Is there a rationale for excluding dissertations/theses from inclusion? 

I wish the authors the best of luck and look forward to reading this review when it has been completed.

Is the study design appropriate for the research question?

Yes

Is the rationale for, and objectives of, the study clearly described?

Yes

Are sufficient details of the methods provided to allow replication by others?

Yes

Are the datasets clearly presented in a useable and accessible format?

Not applicable

Reviewer Expertise:

Childhood speech, language and communication needs; qualitative research; collaborative practice

I confirm that I have read this submission and believe that I have an appropriate level of expertise to confirm that it is of an acceptable scientific standard.

# Peer review: Reviewer response for version 1

**DOI:** 10.21956/hrbopenres.14396.r29351

**Published:** 2021-06-23

**Authors:** Paula Zwozdiak-Myers

**Affiliations:** 1 School of Education, Brunel University London, London, UK

The aims and purposes underpinning the protocol presented for this integrative review of ‘reflective practice across speech and language therapy and education’ have been clearly articulated, soundly argued and justified. Causal factors which may challenge effective co-practice such as reciprocal relationships and power dynamics have been identified as well as some potential solutions e.g., communication processes and the use of reflection to forge new professional identities within novel working contexts. The main aim has been translated into four well-focused research objectives in the quest to determine whether a shared understanding of reflective practice exists between health and education so as to inform the development of a framework for collaborative reflective practice.

 Overall, the research design is appropriate for the purposes intended and draws upon the Preferred Reporting Items for Systematic Review and Meta-Analysis Protocols (PRISMA-P) for guidance which importantly, as the authors acknowledge, does allow for integration of different types of knowledge about practice-based contexts emanating from the diverse literature base which includes theory, policy and empirical studies. There are however some areas which might benefit from further clarification or review by the team:

- Search terms in Table 1 for the ‘sample’ might include initial teacher education and pre-service teachers; and for ‘phenomenon of interest’ might also incorporate collaborative communication and reflexivity given the objectives behind the review.
- In the pursuit to capture a comprehensive and purposeful sample through the search strategy I find it curious that theses (especially doctoral theses given that these must demonstrate Level 8 FHEQ descriptors) are situated within the exclusion criteria…such works do attract significant specialist scrutiny and peer review: many are unpublished but contribute immensely to the grey literature available.
- Although highly appropriate frameworks (e.g., M-MAT and JBI) have been identified to appraise the quality and rigour of the literature under review, particularly in the case of empirical studies and primary research, when appraising ‘the interests of different stakeholders’ and ‘contextualising the positioning of the authors’ I wonder whether Guba and Lincoln’s criteria for establishing ‘trustworthiness of the human instrument’ in qualitative research might add further weight when examining the evidence base.
- There appears to be an anomaly between the paper and Figshare insofar as data synthesis on the checklist excludes any reference of how quantitative data is to be analysed yet the paper makes explicit reference to content analysis, using Nvivo to ‘describe and quantify the phenomenon’ (page 6)…although authors do offer an explanation of how Brookfield’s (1995) Lens theory will initially guide the analysis it is not wholly transparent how the Nvivo computer software is to be used…if not for analysis then perhaps the management of data?

Overall, the paper is scientifically sound and authors have provided sufficient and very explicit details of the methods employed to enable others to assess and replicate the protocol. There are minor lapses in sentence structure which would benefit from further editorial work.

 This is an exciting research enterprise which has great potential to contribute new knowledge to, and practical applications within, the field of speech and language therapists working collaboratively with teachers to ensure inclusion for all our students, and more specifically for those with speech, language and communication needs (SLCN).

Is the study design appropriate for the research question?

Yes

Is the rationale for, and objectives of, the study clearly described?

Yes

Are sufficient details of the methods provided to allow replication by others?

Yes

Are the datasets clearly presented in a useable and accessible format?

Not applicable

Reviewer Expertise:

Reflective practice for professional development in teacher education; Social justice, equity and inclusion; Ethics in educational research

I confirm that I have read this submission and believe that I have an appropriate level of expertise to confirm that it is of an acceptable scientific standard, however I have significant reservations, as outlined above.

# Peer review: Reviewer response for version 1

**DOI:** 10.21956/hrbopenres.14396.r29367

**Published:** 2021-06-09

**Authors:** Lisa Archibald

**Affiliations:** 1 School of Communication Sciences and Disorders, The University of Western Ontario, London, ON, Canada

The proposed study has merit. It focuses on a topic of interest to the field of speech-language pathology & audiology. The initial approach to the question using qualitative methods is appropriate. I did wonder about the emphasis on communication as a way to connect reflection rather than simply a focus on reflection, but this is a very minor point. Just prior to the research aims, the statement about reflective practice in health and education surprised me, only because the project focused on speech-language pathology and education specifically. I did wonder why the project was limited to just these fields, as it seems possible that other professions across health and education may inform thinking about the selection of a reflection framework.

Is the study design appropriate for the research question?

Yes

Is the rationale for, and objectives of, the study clearly described?

Yes

Are sufficient details of the methods provided to allow replication by others?

Yes

Are the datasets clearly presented in a useable and accessible format?

Not applicable

Reviewer Expertise:

language disorder, working memory, practice-based research

I confirm that I have read this submission and believe that I have an appropriate level of expertise to confirm that it is of an acceptable scientific standard.
